# Mesenchymal Stem Cell Exosomes Therapy for Acquired Trichorrhexis Nodosa: A Case Series

**DOI:** 10.1111/jocd.16683

**Published:** 2024-11-25

**Authors:** Xi Chen, Jing Pang, Jianke Li, Xiuhuan Wang, Zihao Mi, Zhenbo Hu, Guoyan Liu

**Affiliations:** ^1^ Hospital for Skin Diseases Shandong First Medical University Jinan Shandong China; ^2^ Shandong Provincial Institute of Dermatology and Venereology Shandong Academy of Medical Sciences Jinan Shandong China; ^3^ Laboratory for Stem Cell and Regenerative Medicine Affiliated Hospital of Shandong Second Medical University Weifang Shandong China

**Keywords:** acquired, exosomes, hair shaft disorder, trichorrhexis nodosa

## Abstract

**Background:**

Recent preclinical studies have demonstrated the potential efficacy of stem cell exosomes in the treatment of hair loss. However, there is a paucity of clinical studies investigating the application of exosomes for this purpose. This case series presents three patients treated with exosomes for acquired trichorrhexis nodosa (ATN), a condition characterized by hair shaft abnormalities and breakage.

**Objectives:**

This study aims to evaluate the effectiveness and safety of utilizing mesenchymal stem cell (MSC) exosomes as a novel therapeutic approach for the management of ATN.

**Methods:**

A standardized process was employed to prepare 0.1 mL of exosomes, which were subsequently injected into bilateral regions of the patients' scalps at 0.5–1 cm intervals on a monthly basis. Each injection comprised a total volume of 5 mL, and all three patients underwent a minimum of four treatment sessions. The comparative efficacy of the treatment was evaluated using clinical photographs, dermatoscopy, and scanning electron microscopy (SEM) for all three patients post‐intervention.

**Results:**

The hair condition of the three patients demonstrated significant improvement, characterized by increased length and density, enhanced pigmentation with a reduced presence of dusty white dots, and the disappearance of dermoscopic black dots and broken hairs. SEM analysis revealed a remarkable recovery in the hair cuticle layers. At the 1‐year follow‐up, hair growth essentially remained normal.

**Conclusion:**

Exosomes derived from mesenchymal stem cells demonstrate efficacy in treating ATN, presenting a novel therapeutic approach for this condition.

## 1 Introduction

Trichorrhexis nodosa (TN), a clinically rare disorder, is characterized by nodular thickening and breakage of hair shafts [1]. Classified as either congenital or acquired, TN presents with one or multiple small nodules under dermoscopy and light microscopy. The hair shaft may fracture at the nodule site, resulting in a stump resembling a broomstick or brush‐like appearance [2, 3, 4]. The diagnostic gold standard for TN is microscopic examination [5]. While primarily affecting the scalp, TN has also been observed in pubic and body hair [6, 7]. Currently, no standardized treatment exists for TN. Exosomes, extracellular vesicles enclosed by a lipid bilayer with diameters ranging from 30 to 150 nm [8], interact with target cells through cytosolic uptake and membrane fusion. This interaction facilitates the transmission of biosignals between cells at both local and remote locations [9]. Exosome therapy has emerged as a promising treatment for alopecia, with substantial evidence supporting its role in promoting follicle proliferation and hair growth [10]. However, there are no reported cases of exosome therapy specifically targeting acquired trichorrhexis nodosa (ATN).

**FIGURE 1:**
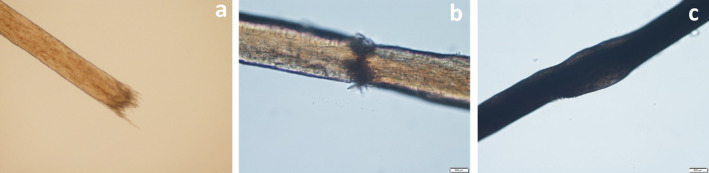
Light microscopy showed “nodular” or “brush‐like” stumps in the hair of three patients (case 1: a, case 2: b, case 3: c).

Three patients presenting at the clinic demonstrated characteristic features of TN, including nodular alterations and brush‐like stumps upon light microscopic examination (Figure 1). Following diagnosis of ATN, these patients received exosome injections as a therapeutic intervention.

## 2 Material and Methods

### 2.1 Preparation of MSC Exosomes

To acquire mesenchymal stem cell exosomes, the culture supernatant was collected when the cells covered 90% of the culture flask bottom. The supernatant was centrifuged at 300 *g* for 10 min to eliminate cell debris and obtain the initial supernatant. Subsequently, the supernatant was centrifuged at 4°C and 2000 *g* for 10 min to remove dead cells and large debris. The resulting supernatant was transferred to a 50‐mL centrifuge tube and centrifuged at 4°C and 10,000 *g* for 30 min to collect the supernatant containing exosome microvesicles. This supernatant was filtered using a 50‐mL syringe connected to a 0.22‐μm filter membrane. The filtered supernatant containing microvesicles was then centrifuged at 70,000 *g* for 60 min to obtain the precipitated exosomes. These exosomes were resuspended in PBS and filtered with a 0.1‐μm filter membrane to achieve high purity. The obtained stem cell exosomes were observed by electron microscopy to assess their size, morphology, and diameter. Western blotting analysis was used to detect the presence of marker proteins on the exosome surface, while nanoparticle tracking analysis was utilized to measure the exosome diameter and particle count. The qualified exosomes were dissolved in physiological saline at a concentration of 1 × 10^10^/mL for injection. MSC exosomes were prepared in the Laboratory for Stem Cell and Regenerative Medicine, Affiliated Hospital of Shandon Second Medical University.

### 2.2 Injection Method

Extract 2.5 mL of exosomes and 0.5 mL of lidocaine injection using a 5‐ml syringe (0.7 × 32 mm TWLB), shaking well to mix the contents thoroughly. Replace the injection needle with a 30G 0.3 × 13 mm needle. Inject the solution subcutaneously, centering the injection at the top of the head and extending to both sides. Position the needle at an angle of approximately 30°–40° to the skin, with the depth of the needle penetrating about 5–7 mm. Administer 0.1 mL of the solution uniformly at intervals of 0.5–1 cm. Repeat the injection process, ensuring the total injected volume reaches 5 mL. This procedure should be performed once a month.

### 2.3 Assessment Method

Clinical photographs, dermatoscopy, and electron microscopy were utilized to compare pre‐treatment and post‐treatment samples from three patients. To ensure an objective evaluation of hair shaft damage severity under electron microscopy, the study employed the 12‐point grading system developed by Lee et al. for scanning electron microscopy (SEM) assessments, which facilitates the assessment of subtle changes in the surface characteristics of damaged hair [11].

## 3 Results

This case series presents three patients with an average age of 28 years (range: 22–38 years) who underwent exosome treatment for TN. None of the patients had a history of similar diseases or other hair abnormalities, with the exception of the father of the patient in case 2, who had a previous diagnosis of alopecia areata. Furthermore, none of the three patients exhibited habits such as frequent hair scratching. Thyroid function and scalp histopathological examination were normal in all cases. Each patient received a minimum of four MSC exosome treatments.

### 3.1 Case 1

**FIGURE 2:**
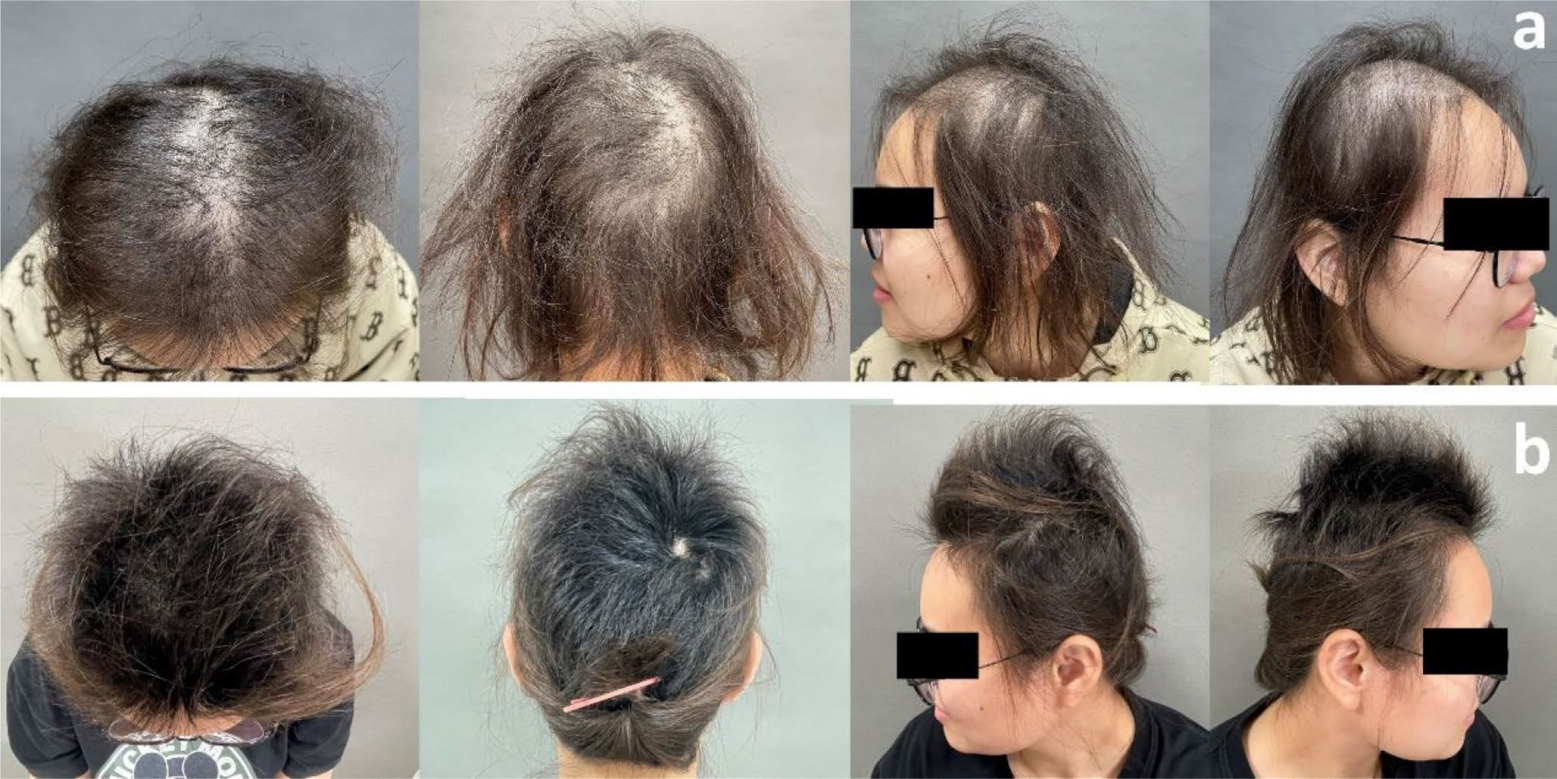
Clinical photographs of Patient 1. (a) Before treatment, the patient exhibited sparse, delicate, and yellowish hairs. (b) Following four treatments, the patient demonstrated reduced hair breakage, darker hair, and increased hair diameter and density.

**FIGURE 3:**
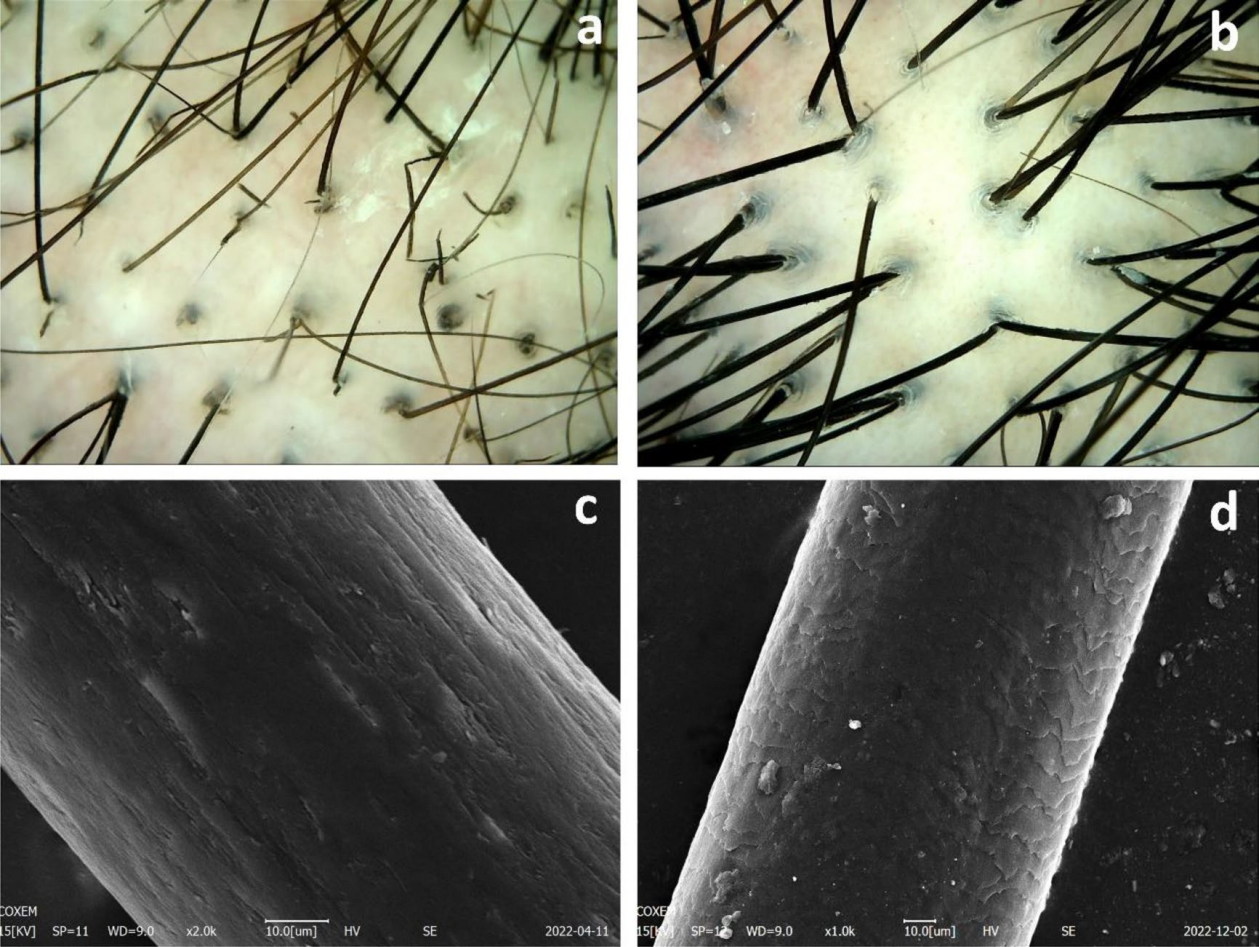
Dermoscopy and SEM findings before and after 4 Treatments of Patient 1. (a) Pre‐treatment dermoscopy (top of head): Visible black dots and hair breakage. (b) Post‐treatment dermoscopy: Hairs return to normal (top of head). (c) Pre‐treatment SEM: Loss of hair cuticle layers. (d) Post‐treatment SEM: Partial reappearance of the hair cuticle layers.

A 24‐year‐old female patient reported increased hair breakage that began following a haircut 3 years prior and remained untreated. In the 6 months preceding the consultation, she observed an escalation in hair loss and heightened susceptibility to breakage. Dermatological examination revealed visibly sparse, fine, and yellowish hairs that were dry with dusty white spots on the shafts, accompanied by increased hair breakage (Figure 2a). Dermoscopy exhibited multiple small nodules and black dots within the hair shafts, as well as visible broken hairs (Figure 3a). SEM demonstrated a loss of hair cuticle integrity and roughness of the shafts; the severity of damage was graded as level 12 according to the 12‐point grading system [11] (Figure 3c). Blood examination results were as follows: hemoglobin, 110 g/L↓; mean erythrocyte volume, 68.2 fL ↓; mean hemoglobin volume, 20.6 ↓; and mean hemoglobin concentration, 301 g/L↓.

After four treatments, there was a noticeable improvement in hair quality. A dermatological examination revealed a significant increase in hair length and density, as well as an enhancement in fine and soft hair texture. Furthermore, the hair texture transformed, becoming denser and darker, while the presence of dusty white dots decreased significantly (Figure 3b). Dermoscopic analysis demonstrated an increased diameter of the hair shafts, accompanied by the disappearance of black dots and broken hairs. SEM further revealed a partial restoration of the hair cuticle layers to their normal state, reaching grade 2 on the 12‐point grading system [11] (Figure 3d).

### 3.2 Case 2

**FIGURE 4:**
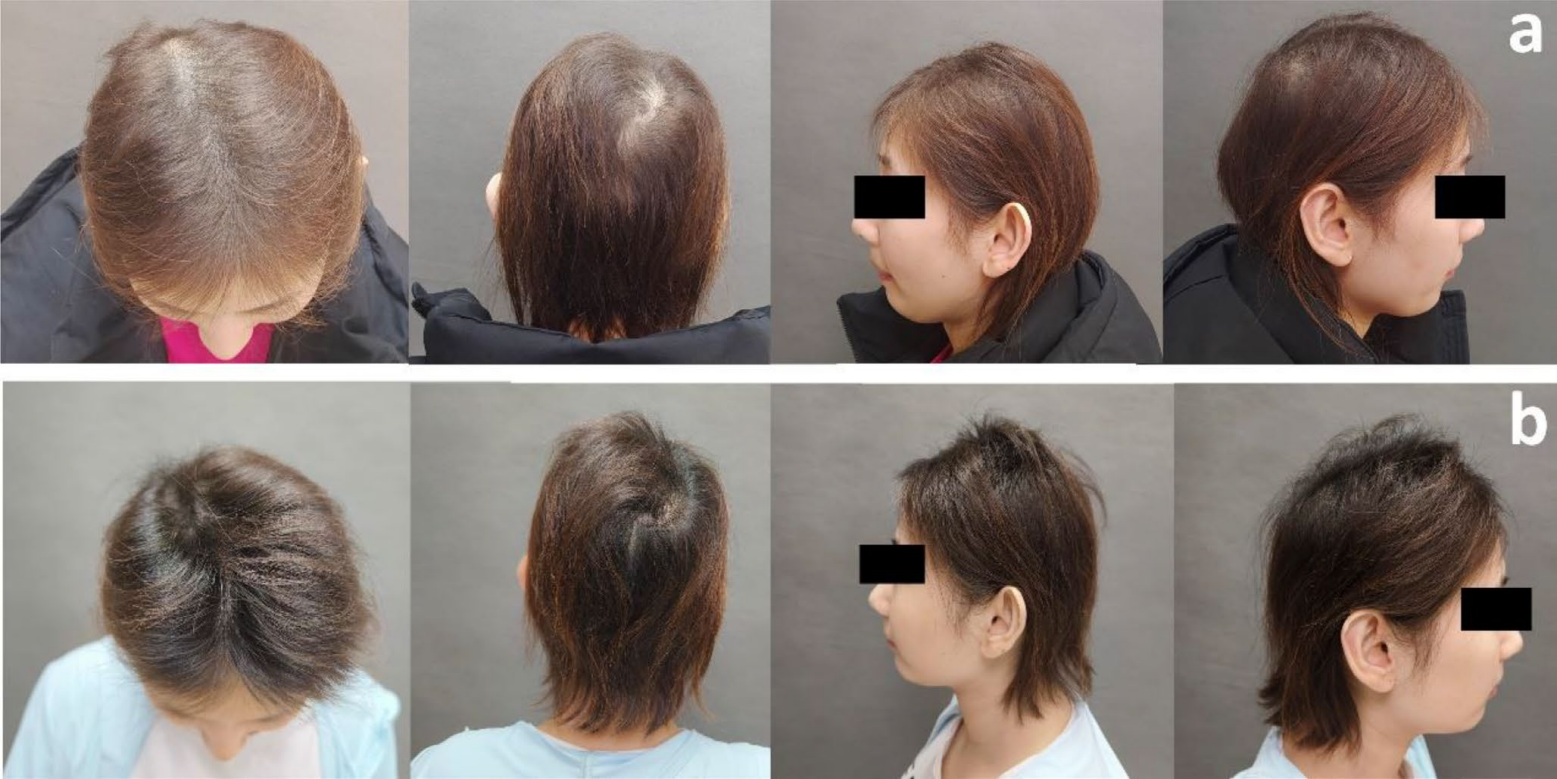
Clinical photographs of Patient 2. (a) Before treatment: Fine, yellowish, dry, and brittle hairs. (b) After six treatments: Increased hair density and improved fine hair.

**FIGURE 5:**
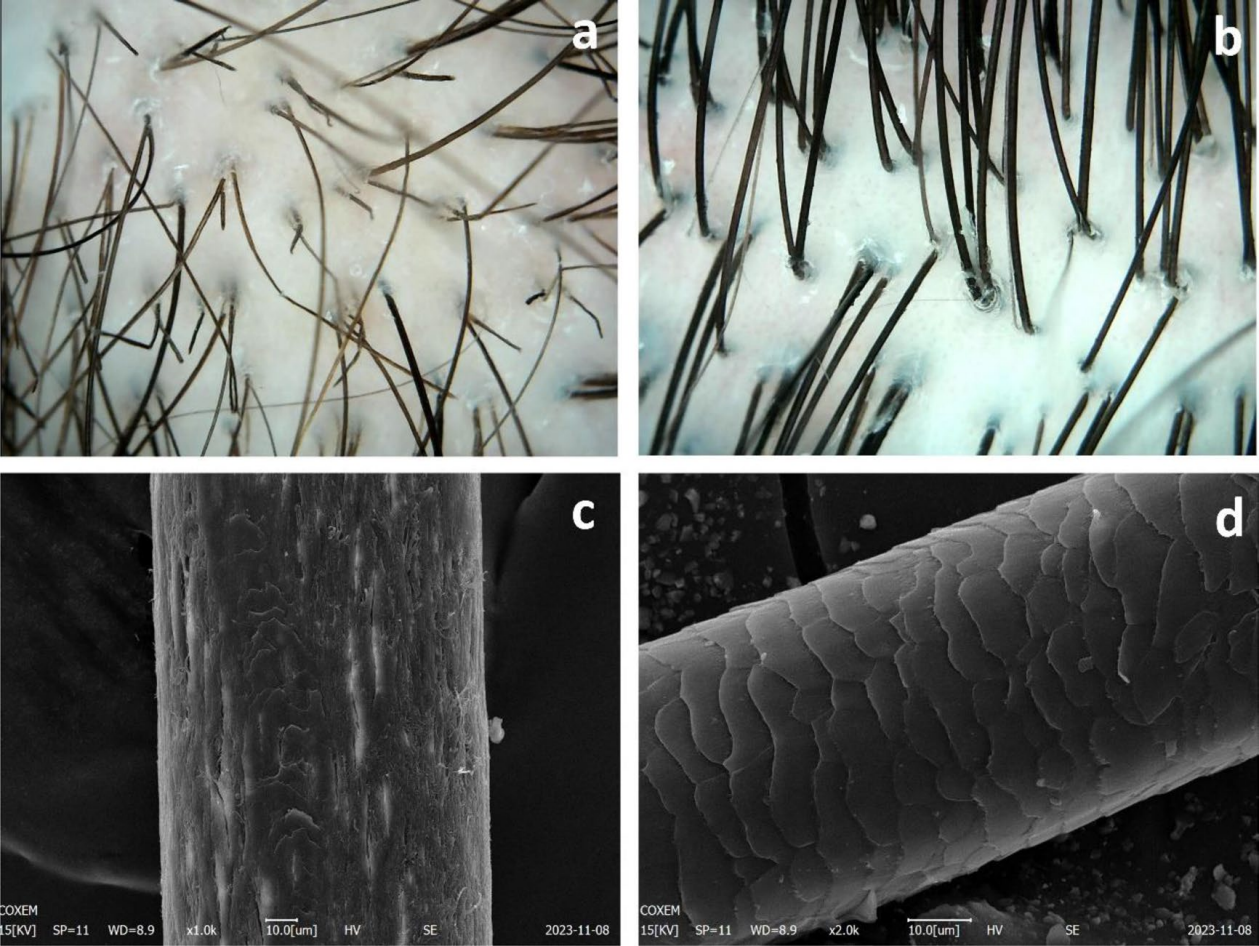
Dermoscopy and SEM findings before and after 6 treatments of Patient 2. (a) Pre‐treatment dermoscopy (top of head): Massive hair breakage. (b) Post‐treatment dermoscopy (top of head): Hair breakage virtually disappeared.(c) Pre‐treatment SEM: Disappearance of the hair cuticle layers and roughness on the surface of the hair shaft. (d) Post‐treatment SEM: The reappearance of the hair cuticle layers.

A 22‐year‐old female patient presented with alopecia areata 3 years prior, exhibiting hair breakage accompanied by dryness and impaired hair growth 2 years ago. These symptoms were exacerbated by a history of hair coloring and previous unsatisfactory outcomes from minoxidil treatment. A dermatological examination revealed fine, yellowish, dry, and brittle hair (Figure 4a). Dermoscopy showed the presence of hair nodules and breakage (Figure 5a). SEM indicated partial loss of the hair cuticle layers, with a severity of grade 11 according to the 12‐point grading system [11] (Figure 5c). Blood examination revealed a hemoglobin level of 74 g/L↓, mean erythrocyte volume of 59.2 fL ↓, mean hemoglobin volume of 15.7 ↓, and a mean hemoglobin concentration of 265 g/L↓.

Following six treatments, dermatological examination revealed increased hair density, improved fine hair texture, darker hair color, and a marked decrease in dusty white dots (Figure 4b). Dermoscopic observation indicated a significant reduction in broken hairs and nodules on the hair shafts (Figure 5b). SEM demonstrated partial restoration of the hair cuticle layers and a return to normal appearance, classified as grade 1 according to the 12‐point grading system [11] (Figure 5d).

### 3.3 Case 3

**FIGURE 6:**
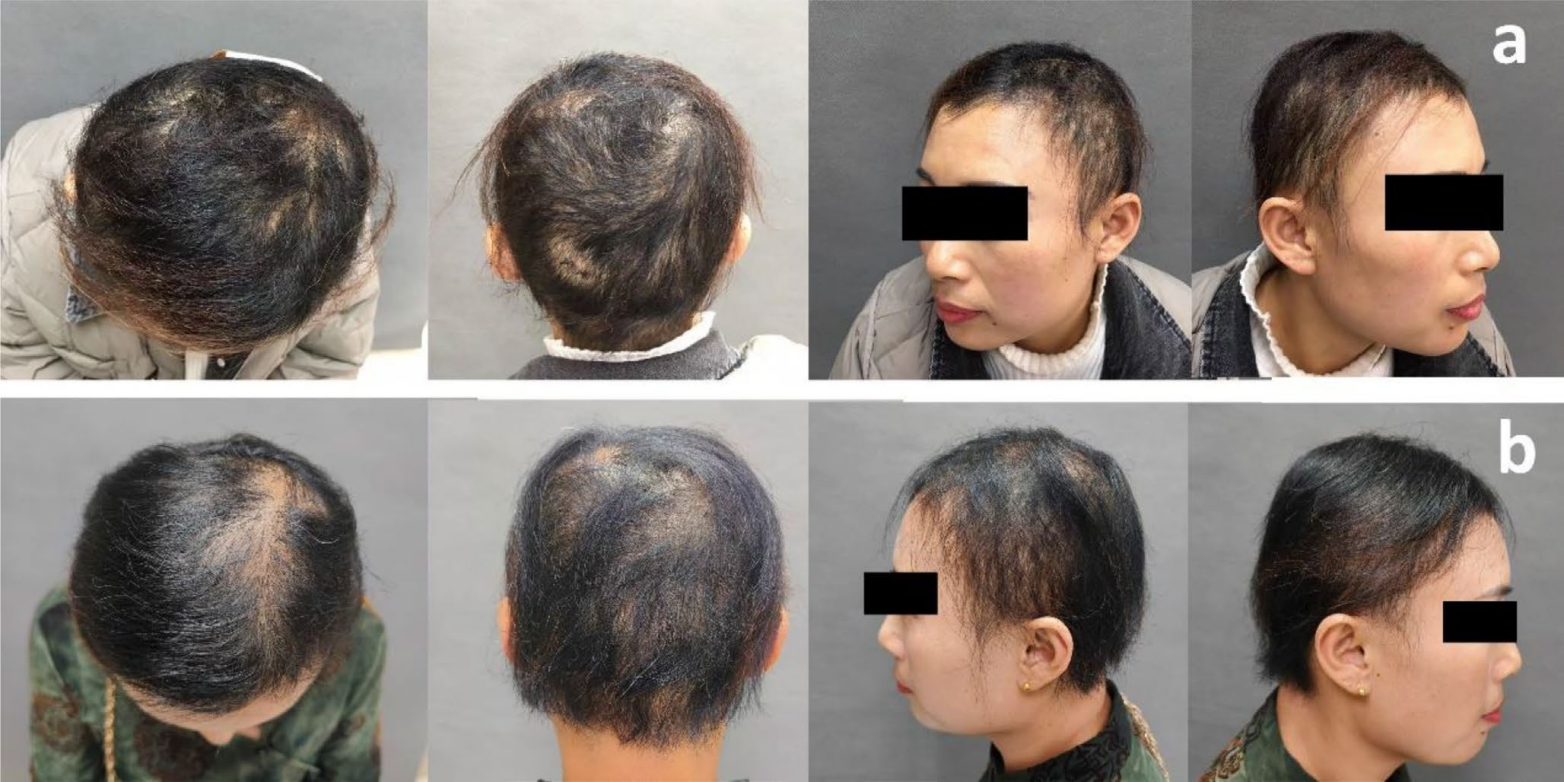
Clinical photographs of Patient 3. (a) Before treatment: Alopecia patches were evident on the scalp, with observable granular white spots on the hair shaft, accompanied by dry and brittle hair. (b) After five treatments: Hair color darkened and the presence of white spots was reduced.

**FIGURE 7:**
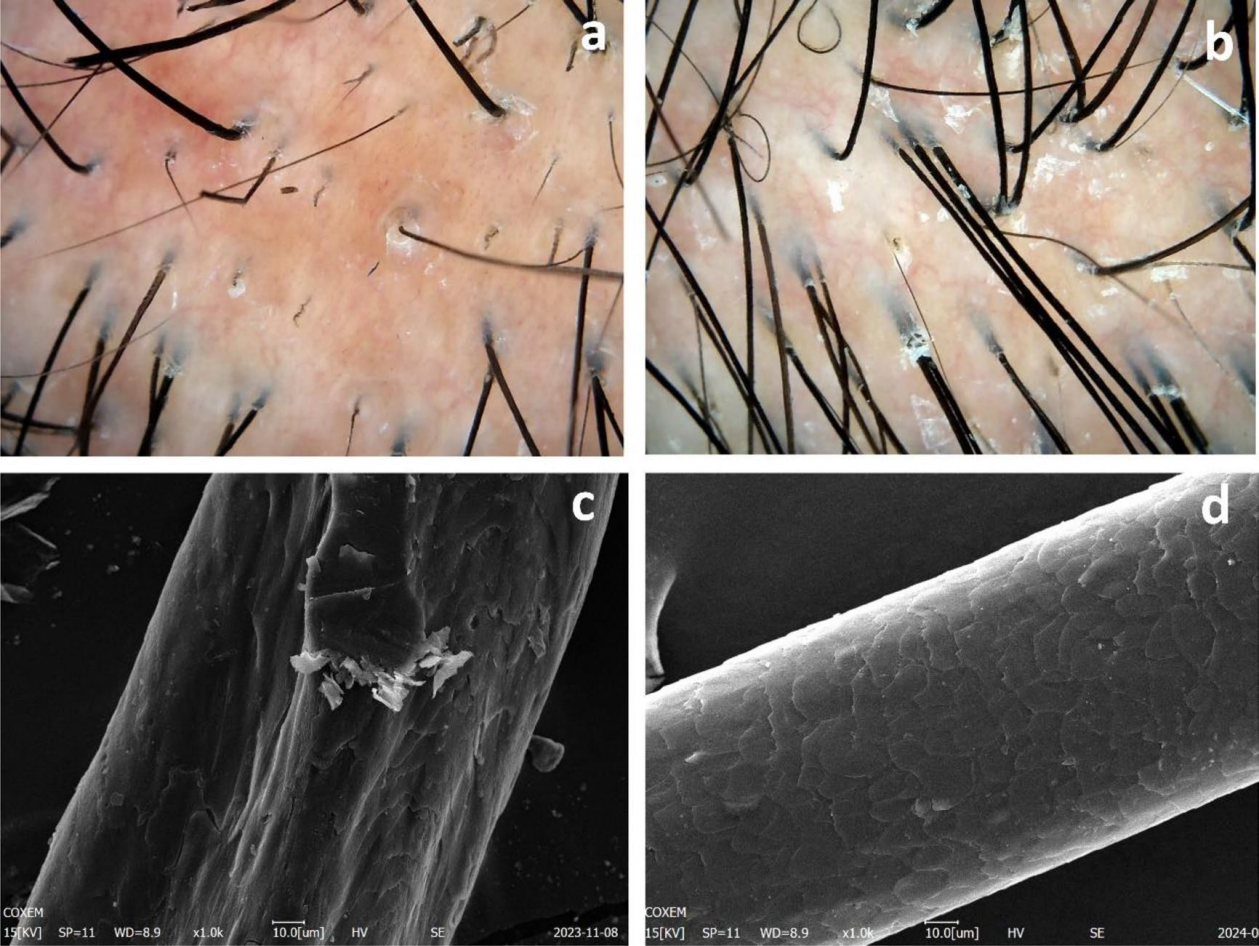
Dermoscopy and SEM findings before and after five treatments of Patient 3. (a) Pre‐treatment dermoscopy (top of head): Scattered black dots and broken hairs. (b) Post‐treatment dermoscopy (top of head): Reduced hair breakage and increased density. (c) Pre‐treatment SEM: Irregular hair shafts and partial loss of the hair cuticle layers. (d) Post‐treatment SEM: The reappearance of the hair cuticle layers.

A 38‐year‐old female patient presented with coronavirus infection, resulting in significant alopecia followed by hair regrowth, some of which exhibited resistance to growth. Previously, an annual occurrence of severe hair loss was observed in April, followed by normal growth. A dermatological examination revealed patchy alopecia on the scalp, white granular dots on the hair shaft, and dry, brittle hair (Figure 6a). Dermoscopy demonstrated broken hairs, cuirasses, and scattered black dots (Figure 7a). SEM revealed irregularities in the hair shaft structure and a partial absence of cuticles in some hairs (Figure 7c). The severity of damage was graded as level 11 according to the 12‐point grading system [11]. Blood examination revealed erythrocytes at 3.65 × 10^9^/L↓, hemoglobin concentration at 98 g/L ↓, erythrocyte pressure at 31.4% ↓, and mean hemoglobin concentration at 312 g/L↓.

Following five treatments, dermatological examination revealed that the hair exhibited a deepened pigmentation and a significant reduction in the presence of white dots (Figure 6b). Dermoscopic analysis revealed an increase in both hair density and diameter (Figure 7b). Furthermore, SEM demonstrated the reappearance of the hair cuticle layers, which partially regained their normal structure (Figure 7d), ultimately resulting in restoration to grade 2 according to the 12‐point grading system [11].

## 4 Discussion

TN can be classified as congenital or acquired. Congenital TN is frequently associated with generalized hair dystrophy and metabolic syndromes, such as biotinidase deficiency or argininosuccinic aciduria [3, 5, 12]. ATN has been linked to various factors, including physical trauma, friction, excessive hair combing, chemical damage from frequent bleaching, and certain systemic diseases, such as iron‐deficiency anemia, hypothyroidism or hyperthyroidism, and malnutrition [5, 7, 13, 14]. However, the underlying mechanism remains unclear. All three patients in this study presented with mild to moderate anemia, suggesting a potential correlation between anemia and ATN that cannot be ruled out. Sakthibalan et al. reported a case of nodular brittle hair caused by severe iron‐deficiency anemia [15]. They proposed that impaired keratin synthesis due to iron deficiency could lead to fragile hair shafts, with neonatal hair returning to normal after treatment with iron supplementation.

Recent preclinical studies have demonstrated promising results for the use of exosomes derived from various cell types in the treatment of alopecia [16]. The development of the hair follicle requires the involvement of a population of follicle‐specific stem cells, including epithelial stem cells, neural crest cells, and mesenchymal stem cells [17, 18]. The rapidly proliferating hair matrices in the hair follicle bulb are responsible for generating the hair shaft. As these matrices differentiate and ascend, they are compressed by the inner root sheath, which shapes the hair. The size and curvature of this shape determine the overall appearance of the hair. The hair papilla, composed of induced fibroblasts located at the base of the hair follicle, is thought to regulate both the number of hair matrices and, consequently, the size of the hair [19]. Liang et al. showed that exosomes derived from adipose stem cells enhance the proliferation, migration, and differentiation of dermal papilla cells (DPCs) while upregulating the expression of cell cycle proteins, β‐linker protein, versican, and BMP2. This leads to the restoration of bulb size and dermal thickness, ultimately promoting normal hair follicle growth [20]. Thus, MSC‐derived exosomes have the potential to increase hair matrix production within the hair shaft by stimulating DPC proliferation, migration, and differentiation. As a result, this leads to an increased number of hair matrices within the hair shaft, improving hair growth and repairing damaged hair shafts in patients with TN.

This study demonstrates the efficacy of exosome therapy in treating patients with ATN. The treatment yielded favorable outcomes, including cosmetic improvement, reduction of hair nodules and hair breakage as observed through dermatoscopy and light microscopy, and restoration of the hair cuticle layers as evidenced by SEM. While previous literature has primarily focused on preventive measures such as avoiding physical or chemical trauma to the hair shaft and treatments targeting the underlying disease [21], there are currently no existing reports on the application of exosome therapy for ATN. Consequently, our study presents a novel treatment option for this condition, offering a promising avenue for further research and clinical application.

## Author Contributions

**Xi Chen:** collection and/or assembly of data, data analysis and interpretation, manuscript writing. **Jing Pang**, **Jianke Li**, **Xiuhuan Wang**, **Zihao Mi:** provision of study material or patient. **Zhenbo Hu:** provision of study material, conception and design. **Guoyan Liu:** collection and/or assembly of data, conception and design, final approval of manuscript, financial support, administrative support.

## Ethics Statement

The study was approved by the Bioethics Committee of the Medical University of Shandong First Medical University, and the treatments were ethically approved by the hospital (ethics no. 20230709KYKTKS002). The patients in this study have given written informed consent to the publication of their case details.

## Conflicts of Interest

The authors declare no conflicts of interest.

## Data Availability

The data that support the findings of this study are available from the corresponding author upon reasonable request.
